# Tailoring stimuli-responsive copolymers *via* a universal poly(glycidyl methacrylate) modification strategy

**DOI:** 10.1039/d6ra06652f

**Published:** 2026-08-14

**Authors:** Sebastian Heinz, Victoria Glößner, Markus Gallei

**Affiliations:** a Saarland University, Polymer Chemistry Campus C4 2 66123 Saarbrücken Germany markus.gallei@uni-saarland.de; b Saarene, Saarland Center for Energy Materials and Sustainability Campus C4 2 66123 Saarbrücken Germany

## Abstract

In this work, glycidyl methacrylate (GlyMA) copolymers have been synthesized together with methyl methacrylate (MMA) as a comonomer *via* living anionic polymerization. Under mild and precisely chosen reaction conditions in tetrahydrofuran (THF) at −78 °C, the ring-opening and crosslinking reaction of the epoxide could be prevented during the polymerization. The polymers were analyzed by size-exclusion chromatography (SEC), nuclear magnetic resonance (NMR) spectroscopy, and Fourier transform infrared (FTIR) spectroscopy, yielding a copolymer with a dispersity of 1.10 and a number-average molecular weight of 25 kDa. After confirming the presence of intact epoxide units, redox-active amines, such as 2-aminoanthraquinone (2AAQ) and 3-ferrocenyl propylamine, were used to functionalize the GlyMA-containing polymer. As an alternative, more effective route, the epoxide ring was opened with an azide as the nucleophile, thereby generating a platform for further copper-catalyzed click reactions in a quantitative manner. Ethynylferrocene and ethynylcobaltocenium hexafluorophosphate were reacted with the azide to yield the corresponding triazole system bearing a redox-active metallocene side chain. Finally, all functionalized polymers with electrochemically addressable groups were then measured by cyclic voltammetry (CV) to assess their electrochemical behavior. The demonstrated strategies show the universal use of GlyMA as a functional monomer for several post-polymerization modifications, combined with the advantages of living anionic polymerization.

## Abstract 2

Living anionic polymerization enabled the synthesis of GlyMA copolymers for versatile post polymerization functionalization with redox-active moieties, yielding electrochemically active materials with tunable properties.

## Graphical abstract

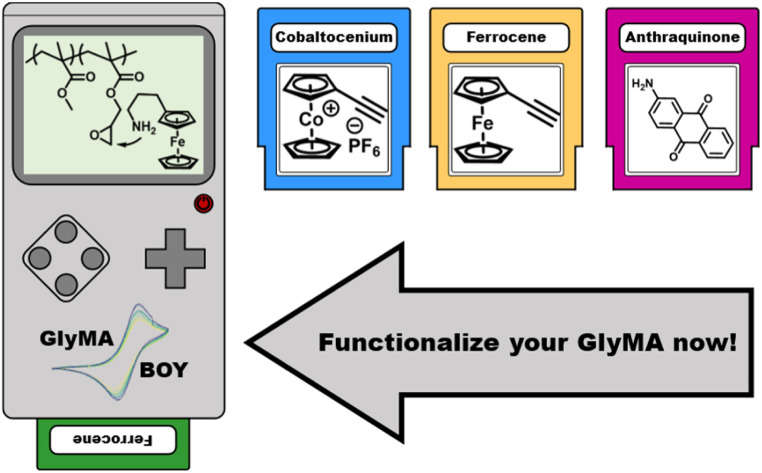

## 1 Introduction

Anionic polymerization is one of the most important controlled polymerization techniques for the synthesis of well-defined macromolecular architectures. In contrast to conventional free-radical polymerization or controlled radical polymerization techniques like reversible-addition–fragmentation-chain-transfer polymerization (RAFT), atom transfer radical polymerization (ATRP), or nitroxide-mediated polymerization (NMP), anionic polymerization proceeds through negatively charged active chain ends, typically carbanions or oxygen anions associated with metal counterions such as lithium, sodium, or potassium. Under strictly controlled purification conditions and in the absence of polymerization-terminating impurities, the process exhibits *living* characteristics, meaning that chain termination and transfer reactions are essentially absent. This, however, imposes severe limitations on the range of compatible monomers and functional groups, as electrophilic centers may react with the active chain end, leading to termination or undesirable side reactions.^1^ Using anionic polymerization results in polymers with predictable molecular weights, narrow molar mass distributions, and complex architectures such as block copolymers (BCP),^2,3^ tapered BCPs,^4^ star polymers,^3^ POM POM structures;^5^ in addition, functionalized chain ends can be obtained.^1,6–9^

Given the limitations of monomers, the polymerization of functional monomers remains a major challenge in anionic polymerization. In particular, monomers containing hydroxyl, amino, accessible carbonyl, or epoxide moieties require carefully optimized reaction conditions or protective strategies to suppress side-reactions and maintain “living” behavior.^10–12^ Despite ongoing research on the synthesis of functional polymers and copolymers in recent years, many challenges regarding functional monomers used in anionic polymerization remain to be overcome, and new strategies need to be developed. Post-polymerization modification of polymers plays a crucial role in producing functional polymers.^13^ A variety of different protocols for post-modification processes in the field of polymers have been applied to introduce functionalities.^13,14^ Powerful polymer post-modification methods for introducing pendant side groups or responsive moieties with additional functionalities into methacrylate-based polymers usually focus on ring opening reactions of epoxides or cyclic anhydrides, transamidation, and transesterification of active esters,^13^ but also strategies like hydrosilylation can be used in order to functionalize such macromolecules.^15^ Besides post-modification strategies, the functional polymers and polymer networks that directly use stimuli-responsive moieties show a broad spectrum of functional groups for various applications.^16,17^

Among functional methacrylate monomers, glycidyl methacrylate (GlyMA) has attracted considerable interest because it combines a methacrylic double bond with a highly reactive epoxide group. The presence of the epoxide moiety enables convenient post-polymerization modification reactions with a wide variety of nucleophiles, including amines, thiols, and alcohols.^18,19^ Thereby making poly(glycidyl methacrylate) an attractive platform for post-modification chemistry providing access to a variety of functional groups, crosslinking reactions and surface immobilization strategies.^20,21^ The use of GlyMA as a post-modification platform is well known in free radical polymerization (FRP), but comes with several drawbacks, such as no controlled chain length, high dispersities and no access to more complex polymer architectures.

Despite these advantages, the anionic polymerization of GlyMA is particularly demanding. The major challenge arises from the intrinsic reactivity of the epoxide ring toward nucleophilic attack. During polymerization, the propagating carbanionic chain end may not only add to the methacrylate double bond but can also attack the epoxide moiety either intramolecularly or intermolecularly.^22^ Such side reactions lead to ring-opening of the epoxide group, resulting in chain transfer, branching, or even irreversible termination of the living chain ends. As a consequence, the preservation of the epoxy functionality during polymerization is difficult and requires strict control over reaction conditions.^23^

The epoxide ring-opening reaction becomes more prone at elevated temperatures or in polar solvents, where the reactivity of the anionic species is higher. Therefore, the anionic polymerization of GlyMA must be carried out at low temperatures and under anhydrous conditions.^22–24^ The choice of counterion and solvent also strongly influences the selectivity between vinyl propagation and epoxide ring-opening, as found out by Michael Szwarc.^25^ Lithium-based initiators are frequently preferred because the tighter ion pairing associated with lithium counterions reduces the nucleophilicity of the propagating species and thereby suppresses undesired attack on the epoxide ring.^11,25,26^ Because of these challenges, the successful anionic polymerization of GlyMA represents an important example of balancing monomer functionality with the extreme sensitivity of living anionic systems. Careful optimization of polymerization parameters enables the synthesis of well-defined epoxy-functional polymers while minimizing side reactions, thereby preserving the reactive epoxide groups for subsequent chemical modification.

**Scheme 1:**
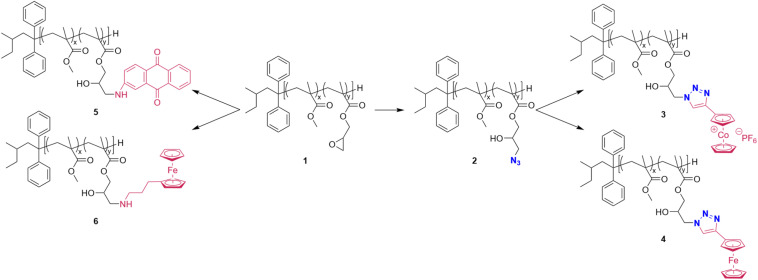
Post-modification strategies of P(MMA-*co*-GlyMA) 1 copolymers in this publication: azide-functionalized copolymer 2 underwent a subsequent click reaction with ethynylcobaltocenium hexafluorophosphate 3 and ethynylferrocene 4. Direct modification was performed using 2-aminoanthraquinone and 3-ferrocenyl propylamine leading to the functionalized and redox-active copolymers 5 and 6.

In this work, the synthesis of P(MMA-*co*-GlyMA) copolymers *via* living anionic polymerization is presented. In addition to the synthesis and characterization of the resulting polymers, several post-functionalization strategies were investigated, with a focus on stimuli-responsive moieties that are difficult to homopolymerize in a controlled fashion. The epoxide groups of the GlyMA units underwent nucleophilic ring-opening reactions with amines such as 3-ferrocenyl propylamine and 2-aminoanthraquinone. Furthermore, to demonstrate the universal applicability of the herein-shown synthetic approach, azide-functionalized polymers were prepared by reaction with sodium azide, thereby enabling subsequent Cu-catalyzed azide–alkyne cycloaddition (“click” reaction) with ethynyl-functionalized ferrocene and cobaltocenium hexafluorophosphate derivatives (Scheme 1). The introduction of redox-active moieties into the polymer backbone yields electrochemically addressable materials that exhibit characteristic responses in cyclic voltammetry. The combination of controlled anionic polymerization with efficient post-polymerization functionalization therefore represents a versatile approach to tailored stimuli-responsive polymer systems, and this route could be translated to pave the way for convenient, tailored design of block copolymers.

## 2 Experimental

### 2.1 Materials

All chemicals were purchased from BLD Pharm, Fisher Scientific, Sigma Aldrich, and TCI chemicals. All chemicals were used as received unless stated otherwise. MMA and GlyMA were prepared according to standard procedures for anionic polymerization, dried over calcium hydride and degassed under vacuum, and stored in the glovebox at −20 °C. Tetrahydrofuran (THF) for anionic polymerizations was degassed, dried using diphenylhexyllithium (DPhLi), and cryo-transferred into a baked-out ampoule before each polymerization. All polymerizations were done under an inert gas atmosphere in the glovebox. Ethynylcobaltocenium hexafluorophosphate was synthesized as described earlier in the literature.^27^ 3-ferrocenyl propylamine was synthesized according to a previously published method.^28^ DMF and toluene were dried over 3 Å molecular sieves. The DMF/water mixtures were degassed by purging with nitrogen gas for 10 minutes.

### 2.2 Instrumentations and characterization

Size-exclusion chromatography (SEC) was performed using DMF as the eluent (1 mL min^−1^ flow rate, containing 1 g L^−1^ LiBr). A PSS GRAM analytical column set from PSS (30 Å, 10^3^ Å, 10^3^ Å) was used at a temperature of 50 °C with a Waters 2410 refractive index detector and a Waters 2487 Dual λ Absorbance Detector. For the SEC using 2,2,2-trifluoroethanol (TFE) with 0.05 M potassium 2,2,2-trifluoroacetate (KTFA) as the mobile phase (1 mL min^−1^), the setup was as follows: two PFG analytical Linear M columns at 30 °C, with a separation range of 1–1 000 000 Da, were used. As a detector, an Agilent Technologies 1260 Infinity G1362 1260 RID and a Waters 2487 Dual λ Absorbance Detector were used. For calibration, PMMA standards from PSS were used. PSS WinGPC^®^ UniChrom V 8.31 was used for data acquisition and evaluation. Results are written with the subscripts describing weight percentages of the monomers and the superscript describing the overall molecular weight of the polymer in kDa. Fourier-transform infrared spectroscopy (FTIR) was performed using an attenuated total reflectance Fourier transform infrared (ATR-IR) instrument from Bruker (Bruker Alpha II) in the range of 400–4000 cm^−1^ with a resolution of 4 cm^−1^. Nuclear magnetic resonance (NMR) spectroscopy was performed using a Bruker Avance II 400 spectrometer with a 9.4 T Ultrashield Plus Magnet and a BBFO probe. The spectra were referenced to the solvent signals. MestReNova 14.0.0 was used to process and evaluate the spectra. Differential scanning calorimetry (DSC) was carried out on a Netzsch DSC 214 Polyma with a heating rate of 10 K min^−1^, nitrogen as both protective and purge gas in flow rates of 60 mL min^−1^ and 40 mL min^−1^, respectively. For evaluation, Netzsch Proteus Thermal Analysis 8.0.1 was used. Thermogravimetric analyses (TGA) were performed using a Netzsch TG 209 F1 Libra with a heating rate of 10 K min^−1^ and nitrogen as both the protective and purge gas at a flow rate of 20 mL min^−1^. For evaluation, Netzsch Proteus Thermal Analysis 8.0.1 was used. Cyclic voltammetry (CV) was performed using a BioLogic SP-150 as the potentiostat in a voltammetry cell with a three-electrode setup. Ag/Ag^+^ as the reference electrode in acetonitrile, a platinum wire as the counter electrode, and a platinum or carbon electrode with an inner diameter of 2 mm. The measurements were conducted in a 0.1 M solution of tetrabutylammonium hexafluorophosphate (TBA[PF_6_]) at a scan rate of 50 mV s^−1^. The evaluation was done using EC-Lab V11.46. For the non-soluble samples, a glassy carbon electrode was drop-coated with a suspension of the polymer and multi-walled carbon nanotubes (2/1; w/w) in chloroform. For the soluble samples, the Pt working electrode was used.

### 2.3 Synthesis

#### 2.3.1 Exemplary anionic polymerization for the preparation of P(MMA_70_-*co*-GlyMA_30_)^25^1

To a heated ampoule containing freshly distilled THF, catalytic amounts of LiCl were added as an additive, and the mixture was stirred with *s*-BuLi (1.3 M, 100 µL) for at least 5 hours at room temperature. A small amount of THF (1.5 mL) was removed as a solvent for the initiator solution. To the ampoule, the monomers 1.49 mL (14.0 mmol, 210 eq.) MMA and 580 µL (4.22 mmol, 63 eq.) GlyMA were added, and the ampoule was cooled to −78 °C. In the syringe containing the THF aliquot, diphenylethylene 17.6 µL (DPE) (100 µmol, 1.5 eq.) was added, followed by 51.3 µL *s*-BuLi (1.3 M, 1 eq., 66.7 µmol, 1 eq). The solution turned dark red, and the syringe was shaken to ensure that all DPhLi had formed. The DPhLi solution was added to the ampoule in a single rapid step to initiate the polymerization. The deep red color of the initiator solution disappeared immediately. After stirring for 5 h, the polymerization was terminated by adding a small amount of degassed methanol to the mixture. After warming up to room temperature, the polymer was precipitated in methanol, filtered off, washed with methanol, and then dried in a vacuum oven at 40 °C overnight.

#### 2.3.2 Anionic polymerization for the preparation of PGlyMA^16^

To a heated ampoule containing freshly distilled THF, catalytic amounts of LiCl were added as an additive, and the mixture was treated with *s*-BuLi (1.3 M, 100 µL) and stirred for at least 5 hours at room temperature. A small aliquot of 1.5 mL of the THF was removed as a solvent for the initiator solution. To the ampoule, 96 µL (0.7 mmol, 212 eq.) GlyMA was added, and the ampoule was cooled to −78 °C. In the syringe containing the THF aliquot, 1.2 µL DPE (6.6 µmol, 2 eq.) and 2.6 µL *s*-BuLi (3.3 µmol, 1 eq.) were added. The syringe was shaken, and the initiator solution was rapidly added to the cooled ampoule containing the GlyMA. After a 4-hours reaction time, the polymerization was terminated by the addition of 100 µL degassed methanol. The polymer was precipitated in methanol, filtered off, washed, and dried under vacuum at 40 °C.

#### 2.3.3 Anionic polymerization for the preparation of P(MMA_61_-*co*-GlyMA_39_)_38_-*b*-PMMA_62_^82^

To a heated ampoule containing freshly distilled THF, catalytic amounts of LiCl were added as an additive, and the mixture was stirred with *s*-BuLi (1.3 M, 100 µL) for at least 5 hours at room temperature. A small aliquot of 1.5 mL of the THF was removed as a solvent for the initiator solution. To the ampoule, 530 µL MMA (4.90 mmol, 122 eq.) and 288 µL GlyMA (2.10 mmol, 51 eq.) were added, and the ampoule was cooled to −78 °C. In the syringe containing the THF aliquot, 10.6 µL diphenylethylene (DPE) (60.2 µmol, 1.5 eq.) was added, followed by 30.8 µL *s*-BuLi (1.3 M, 40.1 µmol, 1 eq). The solution turned dark red, and the syringe was shaken to ensure that all DPhLi had formed. The DPhLi solution was added to the ampoule in a single rapid step to initiate the polymerization. After stirring for 5 h, an aliquot of about 2 mL was taken, terminated using degassed methanol, and precipitated in methanol. The sample was filtered off, washed with methanol, and dried in a vacuum oven at 40 °C overnight. To the reaction mixture, 1.27 mL MMA (12.0 mmol, 293 eq.) was added as the second block, and the polymerization continued. After the mixture was stirred for an additional 2 hours, the polymerization was terminated by adding a small amount of degassed methanol to the mixture. After warming up to room temperature, the polymer was precipitated in methanol, filtered off, washed with methanol, and then dried in a vacuum oven at 40 °C overnight.

#### 2.3.4 Exemplary azide ring-opening-reaction of PGlyMA 2

To a solution of 800 mg P(MMA_70_-*co*-GlyMA_30_)^25^ (240 mg GlyMA, 1.68 mmol, 1 eq.) in dry DMF, 328 mg NaN_3_ (5.04 mmol, 3 eq.) and 270 mg NH_4_Cl (5.04 mmol, 3 eq.) were added, and the mixture was stirred at 50 °C overnight. After the reaction time, the mixture was cooled down to room temperature, and the polymer was precipitated in distilled water. The polymer was then filtered off, washed, and dried under vacuum at 40 °C.

#### 2.3.5 Exemplary click reaction of P(MMA-*co*-HAZPMA) 4

Exemplary click reaction of P(MMA-*co*-HAZPMA) 2 with ethynylferrocene. In a baked-out Schlenk flask, 200 mg P(MMA-*co*-HAZPMA) (0.380 mmol azide, 1 eq.) were dissolved in a mixture of degassed DMF/Water (90/10 by volume). To the mixture 308 mg (1.55 mmol, 4 eq.) sodium ascorbate, 17.4 mg (78.0 µmol, 0.2 eq.) CuBr_2_ and 80 mg (0.380 mmol, 1 eq.) ethynylferrocene were added under an argon atmosphere. The mixture was then stirred for 16 h at room temperature. The reaction mixture was filtered through a 0.45 µm PTFE syringe filter to remove any undissolved components, and the polymer was precipitated in distilled water and centrifuged. The supernatant was poured off, the polymer was dissolved in DMF, precipitated with water, and then centrifuged again. This step was repeated until the supernatant liquid was clear and colorless. The polymer was then dried at 40 °C under vacuum overnight. For the click reaction with ethynylcobaltocenium hexafluorophosphate, 136 mg (0.380 mmol, 1 eq.) was used to yield copolymer 3. For precipitation, ethyl acetate was used.

#### 2.3.6 Ring-opening of PGlyMA with 2-aminoanthraquinone 5

200 mg of P(MMA_70_-*co*-GlyMA_30_)^25^1 (60 mg GlyMA, 0.42 mmol, 1 eq.) were added to a baked-out 2-neck flask equipped with a condenser and a rubber septum. 20 mL of dry dimethylformamide (DMF) was added under argon, and the mixture was stirred until the polymer was fully dissolved. 468 mg (2.1 mmol, 5 eq.) of 2-aminoanthraquinone 7 (2-AAQ) were added to the mixture, the rubber septum was replaced with a glass stopper, and the mixture was heated to 105 °C for 3 days. After cooling down to room temperature, the dark red mixture was concentrated to about 10 mL, and 20 mL THF was added. The mixture was then slowly poured into *n*-hexane under strong stirring. The precipitated polymer was collected and dissolved in THF. After another precipitation in *n*-hexane, the polymer was centrifuged off. The orange supernatant liquid was discarded, and the polymer was dissolved in THF, precipitated in *n*-hexane, and centrifuged again. This step was repeated until the supernatant liquid was colorless. The residual polymer was then dried under vacuum at 40 °C to yield a pale red solid.

#### 2.3.7 Ring-opening reaction of PGlyMA using 3-ferrocenyl propylamine 6

In a baked-out 2-neck flask fitted with a condenser and a rubber septum, 200 mg P(MMA_70_-*co*-GlyMA_30_)^25^1 (60 mg GlyMA; 0.42 mmol, 1 eq.) were dissolved in 20 mL dry toluene under an argon atmosphere. 510 mg (2.1 mmol, 5 eq.) 3-ferrocenyl propylamine 8 were dissolved in a small amount of dry toluene and added to the reaction flask through the septum. The septum was replaced with a glass stopper, and the reaction was stirred for three days at 90 °C. After cooling down to room temperature, the polymer was precipitated in *n*-hexane. The mixture was centrifuged, and the supernatant liquid was poured off. The polymer was washed with *n*-hexane and centrifuged until the supernatant liquid was colorless. The product was then dried at 40 °C in a vacuum overnight, yielding a dark red solid.

## 3 Results and discussion

### 3.1 Synthesis of P(MMA-*co*-GlyMA) copolymers and P(MMA-*co*-GlyMA)-*b*-PMMA block copolymers

**Scheme 2:**
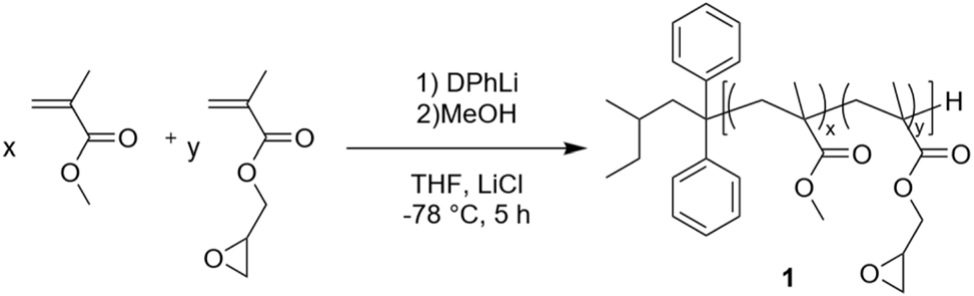
Preparation of P(MMA*-co*-GlyMA) 1*via* anionic polymerization in THF at −78 °C, using diphenylhexyl lithium as an initiator and lithium chloride as an additive.

The living anionic polymerization of glycidyl methacrylate (GlyMA) remains a challenge because the epoxide side chain acts as an electrophilic center that competes with the double bond during propagation. With diphenylhexyl lithium (DPhLi) as an initiator and LiCl as an additive, P(MMA-*co*-GlyMA) copolymers were successfully obtained in THF at low temperatures (Scheme 2).

**Fig. 1:**
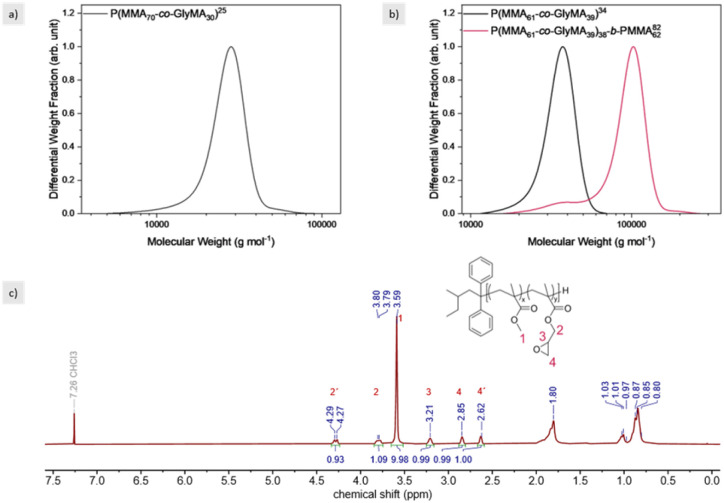
Molar mass distribution derived by SEC of P(MMA_70_-*co*-GlyMA_30_)^25^1 (a) and P(MMA_61_-*co*-GlyMA_39_)_38_-*b*-PMMA_62_^82^ (b), measured in dimethylformamide (DMF) against PMMA standard and the corresponding ^1^H-NMR spectrum of P(MMA_70_-*co*-GlyMA_30_)^25^1 (c).

After precipitation and drying, the polymer was analysed *via* SEC (measured in DMF *vs.* PMMA standards). A number-average molecular weight of 25.0 kDa was determined, with a dispersity *Ð* of 1.10, corresponding to the targeted molecular weight (Fig. 1a). This shows that anionic polymerization can yield PGlyMA copolymers with well-defined molecular weights and molar mass distributions. The low dispersity index also indicates that no crosslinking of the epoxide groups occurred during polymerization, and that the epoxide groups remain intact. Before proving the presence and activity of the epoxide functionality, BCPs featuring GlyMA will also be described. The anionic polymerization of a P(MMA-*co*-GlyMA)-*b*-PMMA BCP was performed stepwise, using GlyMA and MMA for the first block segment and pure MMA for the second. The SECs of the first and final block copolymers are shown in Fig. 1b. A shift in molecular weight between the two blocks is observed, with the first block in black showing a number average molecular weight of 34 kDa (*Ð* = 1.06) and the corresponding BCP in red showing 82 kDa (*Ð* = 1.14), both measured in DMF against PMMA standards. Only a small portion of the first terminated block segment was visible in the SEC diagram of the BCP, accounting for about 7 mass% of the final BCP. This could be due to a few impurities during the addition of the second monomer, MMA. Furthermore, the shift shows the ability of the P(GlyMA-*co*-MMA) to react further *via* the active macroanion and to propagate upon the addition of the new monomer, MMA. To obtain more information on the polymer composition, ^1^H-NMR spectroscopy was performed (Fig. 1c). Despite the peak at 3.59 ppm of the CH_3_ group of the MMA unit, the NMR spectrum showed five peaks corresponding to the epoxide functionality of the GlyMA. The CH_2_ unit next to the carbonyl moiety gives two peaks at 3.8 ppm and 4.28 ppm as a racemic mixture of the monomer is used. The proton of the CH unit of the epoxide ring can be observed at 3.21 ppm. The CH_2_ group of the epoxide ring again shows two different signals at 2.62 ppm and 2.85 ppm due to the stereogenic center of the GlyMA.^29^ By calculating the composition of the copolymer from the NMR spectrum, a composition of 70 mass% MMA and 30 mass% GlyMA is determined. This matches the targeted composition of the anionic polymerization. As an optical method to visualize intact epoxide rings after polymerization, the Preussmann test was used. In the presence of epoxides, the Preussmann reagent causes a color change from white to purple (Fig. S1).^30–33^

**Fig. 2:**
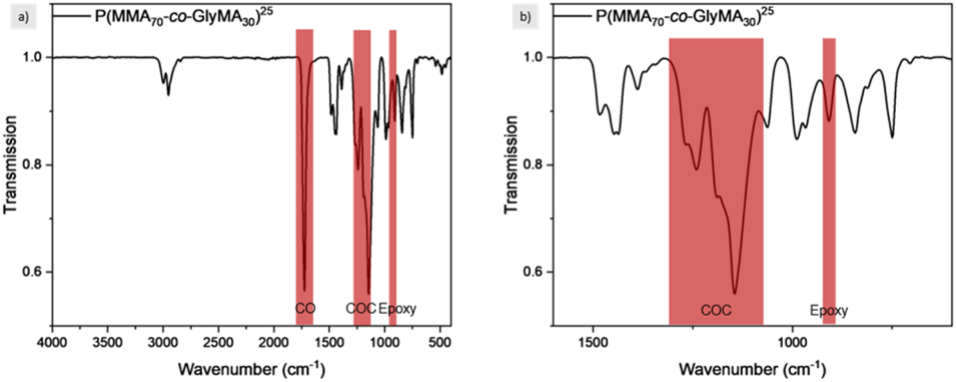
Fourier transform infrared (FTIR) spectrum of P(MMA_70_-*co*-GlyMA_30_)^25^1 (a), showing the vibrations of the methacrylic backbone and the epoxide functionality at 908 cm^−1^ with zoomed area (b).

Results of the FTIR analysis of the P(MMA_70_-*co*-GlyMA_30_)^25^1 copolymer is shown in Fig. 2. The valence vibration of the epoxide groups is assigned to the signal at 908 cm^−1^. The C=O stretching vibration and the C–O–C stretching vibration of the methacrylic unit can be observed at 1724 cm^−1^ and 1390 cm^−1^, respectively. PGlyMA was also polymerized as a homopolymer. Using LiCl as an additive and DPhLi as an initiator, the polymerization was done at −78 °C under the same conditions as the copolymerization and block copolymerization. A number average molecular weight of 30 kDa was targeted, despite the SEC data shown in Fig. S2 and ^1^H-NMR spectrum (Fig. S3) in the SI, which suggests a number average molecular weight of 16.2 kDa with a dispersity of 1.44. Combined with a yield of 4.8 mass%, the deviation between the targeted and achieved molecular weights suggests that homopolymerization of GlyMA alone is a particularly challenging process, accompanied by diverse side reactions. By combining GlyMA with other methacrylate-based monomers such as MMA, the polymerization process gains control, and the advantages of anionic polymerization can be imparted to the resulting polymers.

### 3.2 Direct introduction of redox-active moieties *via* amine ring-opening reaction of the epoxide group

**Scheme 3:**
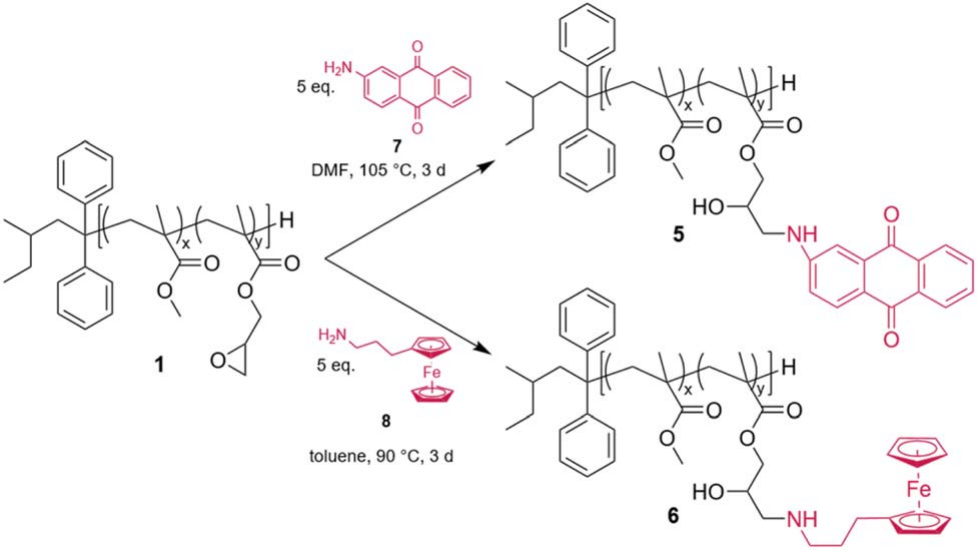
Ring-opening reaction of GlyMa using different amine-containing nucleophiles, with 2-amino anthraquinone 7, in dimethylformamide (DMF) at 105 °C for 3 days and with 3-ferrocenyl propylamine 8 in toluene at 90 °C for 3 days.

Using amines as nucleophiles to open the epoxide ring of glycidyl methacrylate is a widely spread method to bring functionalities into the polymer.^18^ Here, we first focus on the implementation of redox-active moieties, 2-aminoanthraquinone 7 (2-AAQ) and 3-ferrocenyl propylamine 8, to functionalize the P(MMA-*co*-GlyMA) copolymer. The ring-opening reaction using 2-AAQ 7 was carried out in DMF at 105 °C for 3 days, using 5 equivalents of amine relative to the epoxide groups (Scheme 3). Using 3-ferrocenyl propylamine as the nucleophile, the reaction was carried out in toluene at 90 °C for 3 days with 5 eq. of the amine with respect to the epoxide groups (Scheme 3).

**Fig. 3:**
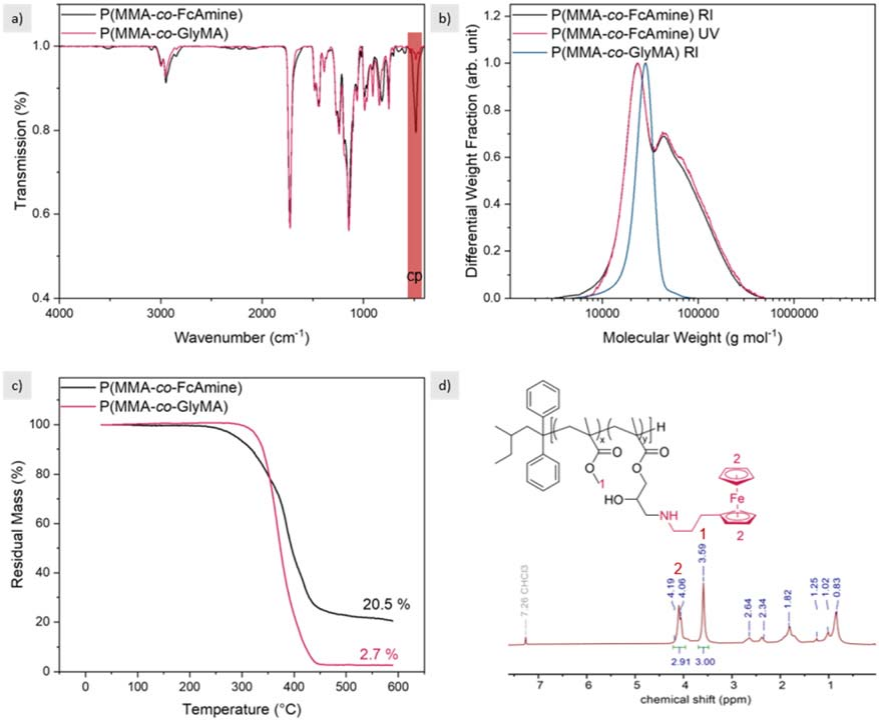
Fourier transform infrared spectroscopy-spectrum (FTIR) of P(MMA-*co*-FcAmine) 6 (a), SEC traces of the UV and RI detector measured in dimethylformamide *vs.* PMMA compared to the original unmodified polymer (b), TGA measurement in nitrogen atmosphere (c) and ^1^H NMR spectrum measured in CDCl_3_ at 400 MHz (d).

2-Aminoanthraquinone 7 features the amino group in direct proximity to the aromatic system, which reduces the nucleophilicity of the nitrogen's free electron pair.^34^ After stirring the mixture for 3 days at 105 °C in DMF and precipitation in *n*-hexane with subsequent washing, the white powder has turned into a pale red solid, giving a first sign that the 2-AAQ reacted with the epoxide. The SEC data measured in DMF against PMMA standards (Fig. S4a) showed a number average molecular weight of 27.1 kDa with a dispersity of 1.29. Additionally, a UV signal is visible, consistent with the RI signal of the copolymer; in the SEC measurement of the unmodified copolymer, no UV signal was observed. The ^1^H-NMR spectrum (Fig. S4d) does not show any aromatic signals of the anthraquinone unit. Comparing the ratio between the signal of the CH_3_ corresponding to the MMA and the signals resulting from the intact epoxide ring with the ratio of the signals of the copolymer before the reaction (Fig. 1c), a difference is observed. Whereas the unmodified copolymer has a ratio of MMA/GlyMA of 9.98, the copolymer after the reaction shows a ratio of 12.78, which means the epoxide groups with respect to the CH_3_ group from the MMA became less, which means about 21.8% of the epoxides opened by the 2-AAQ addition. This low degree of functionalization could be attributed to the low nucleophilicity of the nitrogen electron pair, as it is in direct proximity to the aromatic system. This could explain the absence of aromatic signals from 2-AAQ, as 21.8 mol% of the GlyMA are functionalized, whereas this block accounts for only 30 mass% of the whole copolymer, resulting in a low overall functionalization. This low degree of functionalization has been described in the literature, where GlyMA was incorporated into the shell of a core–shell particle system, with further functionalization of the epoxide groups with 2-AAQ.^33^ Further optimization of the reaction could be done by adjusting reaction time and temperature. A small linker between the amino group and the anthraquinone system could increase the nucleophilicity of the amine. Comparing the TGA (Fig. S4b) and DSC (Fig. S4c) data of the functionalized polymer with the pristine one, a residue of 10.0 mass% at 600 °C under a nitrogen atmosphere was observed, which is 7.3% higher than that of the unfunctionalized one. This indicates that the 2-AAQ is present in the copolymer, leading to a higher residual mass. The glass transition temperature changes from 116.8 °C for the unmodified sample to 121.4 °C for the 2-AAQ sample. This increase in the glass transition temperature suggests that the polymer became more rigid, possibly due to the anthraquinone units bound to it. After the ring-opening of the GlyMA using the 2-AAQ, which resulted in a low degree of epoxide opening, another reaction was tested. 3-Ferrocenyl propylamine 8, as the nucleophile for ring-opening of the epoxide, yields a metallopolymer with the redox-active ferrocene units in the side chain.^35–38^ The FTIR spectra in Fig. 3a show the vibrations of the cyclopentadienyl ring at 495 cm^−1^, indicating the presence of ferrocene in the polymer, with the intensity of the epoxide signals reduced. The hydroxy group formed during the reaction shows a broad, low-intensity signal at 3513 cm^−1^. The SEC (Fig. 3b) shows a strong UV signal matching the signal of the RI detector, whereas the unmodified polymer shows no UV signal, with the RI signal shown in Fig. 3b. Concluding that ferrocene is bound to the polymer. A broad shoulder at higher molecular weights, adjacent to the original peaks, is visible. The shoulder could result from an inhomogeneous incorporation of ferrocene into the copolymer, leading to a higher number-average molecular weight of 31.1 kDa and a dispersity of 1.8. The SEC trace also shows a broadening to lower molecular weights compared to the original polymer. This is due to changes in hydrodynamic volume and potential collapse of the polymer chain upon incorporation of the ferrocene unit.^39^ In addition, SEC is a relative method, and the samples are measured against a PMMA standard that differs greatly from the actual polymer, which may lead to lower molecular weights being observed after modification.^39^ By introducing the metal center of the ferrocene unit into the copolymer, the residue after heating to 600 °C under a nitrogen atmosphere increases to 20.5 mass% compared to 2.7 mass% for the unmodified copolymer (Fig. 3c). Literature suggests that the residue of the ferrocene unit is composed of elemental iron and iron oxide species.^40–42^ For the unmodified polymer, the resonance signals of the aromatic unit of the ferrocene show a broad signal at 4.06 ppm in the ^1^H-NMR spectrum. The peaks of the epoxide unit of the original GlyMA monomer at 2.62, 2.85, 3.21, 3.80, and 4.28 ppm are no longer visible, whereas the peak of the CH_3_ of the MMA still remains at 3.59 ppm, indicating that the epoxide was opened by the amine (Fig. 3d).

**Fig. 4:**
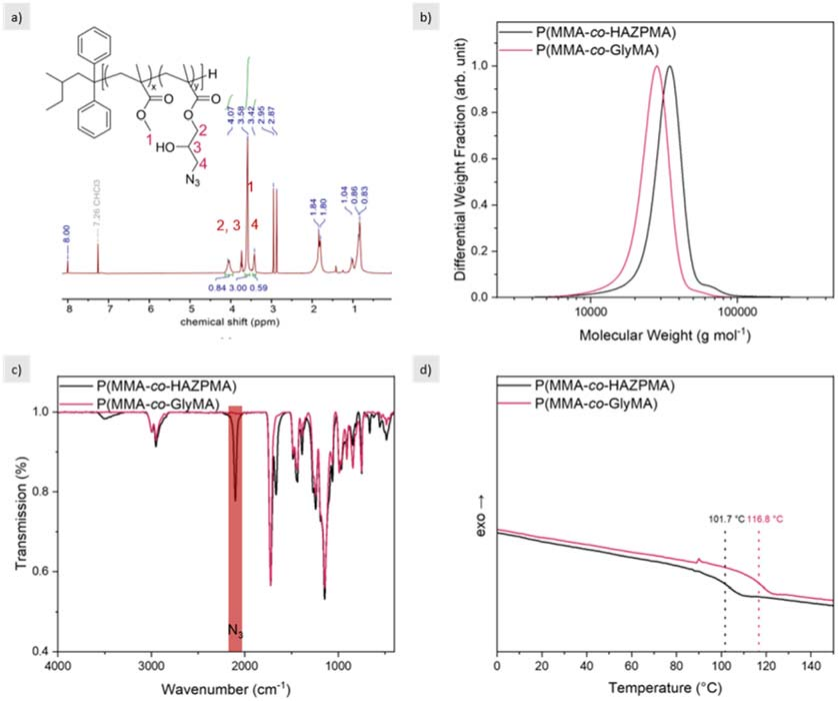
^1^H-NMR spectrum of P(MMA-*co*-HAZPMA) 2 measured in CDCl_3_ showing the opening of the epoxide groups (a), SEC data of the RI detector of P(MMA-*co*-GlyMA) 1 and P(MMA-*co*-HAZPMA) 2 measured in DMF against PMMA standard. Indicating the increase in molecular weight of the polymer after the azidation of the epoxide groups (b). FTIR-spectrum of P(MMA-*co*-HAZPMA) 2. The vibration of the azide group can be observed at 2102 cm^−1^ (c), DSC thermogram of P(MMA-*co*-GlyMA) 1 and P(MMA-*co*-HAZPMA) 2 (d).

#### 3.2.1 Azide-mediated ring-opening and universal post-modification *via* click-reaction

**Scheme 4:**
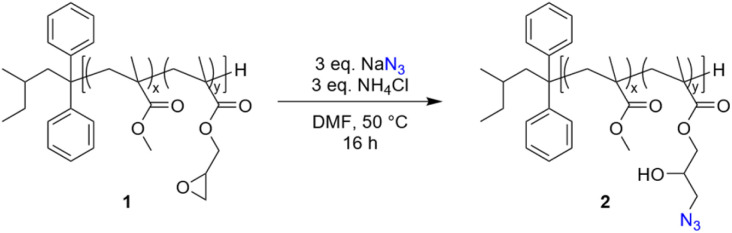
Ring-opening of the epoxide group of P(MMA_70_-*co*-GlyMA_30_)^25^1 using sodium azide, introducing the azide functionality yielding polymer 2.

Azides play a significant role in organic synthesis, providing a pathway for further modifications.^13,18^ They enable click chemistry, in which an azide compound reacts with an alkyne to form a triazole. In our study, the epoxide ring of GlyMA serves as an electrophilic center for ring-opening with an azide as the nucleophile. This azide then provides the platform for the copper(i)-catalyzed azide–alkyne cycloaddition (CuAAC) with different redox-active molecules bearing an alkyne moiety. Using NaN_3_ as a nucleophile to open the epoxide ring of glycidyl methacrylate, with ammonium chloride as a proton donor, has already been reported by the Matyjaszewski group.^43^ With DMF as a solvent and 3 equivalents of both sodium azide and ammonium chloride, the reaction mixture was stirred at 50 °C overnight (Scheme 4), leading to the formation of 2-hydroxy-3-azidopropyl methacrylate (HAZPMA). As the azide group does not feature any protons, the group itself cannot be observed in the ^1^H-NMR spectrum. However, the epoxide ring-opening reaction causes the GlyMA signals to change, appearing at 4.07 ppm (CO_2_C*H*_2_ and C*H*–OH) and 3.42 ppm (C*H*_2_N_3_) (Fig. 4a). The additional signals at 8.00, 2.95, and 2.87 ppm correspond to small amounts of residual DMF from the reaction. As the previous signals from the epoxide group had completely disappeared, the NMR spectrum indicated that the reaction had undergone full conversion. During the reaction, no branching or crosslinking was observed, as indicated by the SEC traces of the polymer (Fig. 4b), with a small increase in dispersity from 1.10 to 1.14. The number average molecular weight also increased to 30.6 kDa, as evidenced by a shift of the signal toward higher molecular weights in the SEC traces. The N_3_ group shows a strong, sharp signal in the FTIR spectrum at 2102 cm^−1^ corresponding to its valence vibrations, which is not present in the unmodified polymer (Fig. 2), and the OH group, which is formed from the oxygen of the former epoxide, shows a broad signal at around 3500 cm^−1^ (Fig. 4c). The glass transition temperature decreases by 15.1 °C to 101.7 °C, as shown in the DSC thermogram in Fig. 4d. The lower *T*_g_ could be explained by the opened epoxide being more flexible, allowing greater movement of the polymer than the rigid ring system did before. As no signal of the original GlyMA unit is visible in the NMR spectrum, a full conversion to the azide can be assumed. Taking the number-average molecular weight of 25 kDa for the original P(MMA_70_-*co*-GlyMA_30_)^25^1, the new theoretical molecular weight of the polymer can be calculated using the new molecular mass of the HAZPMA (185.18 g mol^−1^). By this calculation, the polymer would now be labeled as P(MMA_64_-*co*-HAZPMA_36_)^27^2. The calculated overall molecular weight of the copolymer is slightly lower than that measured by SEC. This difference is due to the SEC being a relative method using a PMMA standard, which differs from the measured polymer. The results presented and discussed above show that the ring-opening of glycidyl methacrylate-containing copolymers using the azide anion as a nucleophile is a highly efficient and reliable method for functionalizing the novel copolymers prepared herein. The resulting polymers provide a universal approach to introducing stimuli-responsiveness by incorporating functional organic molecules, a feature often not accessible *via* direct polymerization of functional monomers. Therefore, the click reaction with various functional alkynes will be discussed in the following section.

### 3.3 Click reaction using ethynylcobaltocenium hexafluorophosphate 9

**Scheme 5:**
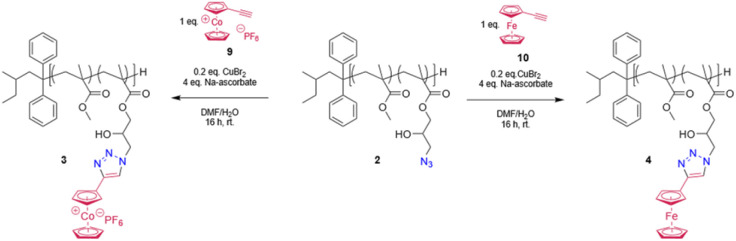
Copper click reactions of P(MMA-*co*-HAZPMA) 2 with ethynylcobaltocenium hexafluorophosphate 9 and ethynylferrocene 10. Resulting in the corresponding modified copolymer with the azide being part of the triazole ring system.

**Fig. 5:**
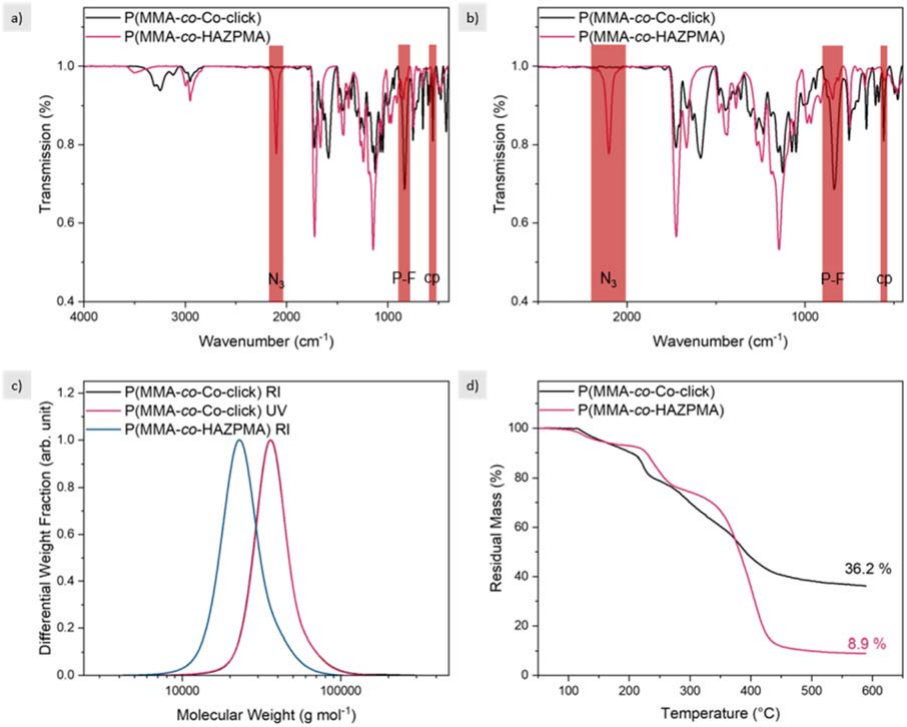
FTIR-spectrum of the copper click reaction using the ethynylcobaltocenium hexafluorophosphate 9 (a) with zoomed area (b), SEC measurements in TFE against PMMA standard (c) and TGA measurement under nitrogen atmosphere (d).

The Cu(i)-catalyzed click reaction is a versatile method for synthesizing triazoles from azides and alkynes under mild reaction conditions.^44–46^ As the first functional alkyne in the present study, we used ethynylcobaltocenium hexafluorophosphate 9, as previous studies have shown that the compound exhibits good PFAS absorption properties and interesting electrochemical behavior.^27,47,48^ The click reaction (Scheme 5) was performed in a mixture of degassed DMF and water (90/10; V/V). As a copper source, 0.2 eq. (in respect to HAZPMA units) CuBr_2_ was used and was *in situ* reduced to the active copper(i) species by 4 eq. of sodium ascorbate. Ethynylcobaltocenium hexafluorophosphate was used in stochiometric amounts. After the reaction was stirred at room temperature overnight, the mixture was precipitated in ethyl acetate and washed multiple times; the polymer was then dried at 40 °C under vacuum. As can be concluded from the corresponding IR spectra in Fig. 5a and b, the azide peak of the educt at 2102 cm^−1^ is no longer visible, as the azide is now part of the triazole moiety. The aromatic cyclopentadienyl system and the PF6^−^ counterion of the introduced cobaltocenium show signals at 557 cm^−1^ (cp) and 834 cm^−1^ (P–F), indicating that the click reaction was successful. As the resulting polymer was no longer soluble in DMF after drying, SEC was measured in 2,2,2-trifluoroethanol (TFE) against a PMMA standard. The SEC shown in Fig. 5c shows the UV and RI signal of the cobalt functionalized polymer in comparison to the P(MMA-*co*-HAZPMA), which was also measured in TFE. A shift of the SEC trace to a higher molecular weight is observed, accompanied by a strong signal from the UV detector, indicating that the aromatic system is bound to the polymer and the reaction. The number average molecular weight of the polymer after the click reaction increased to 35.5 kDa, an increase of 11.8 kDa relative to the P(MMA-*co*-HAZPMA) bearing the azide group. A monodisperse curve is observed with a dispersity of 1.10, indicating a narrow distribution even after the click reaction. The SEC data of the unmodified P(MMA_70_-*co*-GlyMA_30_)^25^ measured in TFE are shown in Fig. S5 in the SI.

With the introduction of the cobaltocenium unit, the ceramic yield after burning the sample at 600 °C in a nitrogen atmosphere increased markedly to 36.2 mass% compared to 8.9 mass% for the azide-functionalized polymer (Fig. 5d). Degradation starts at 116 °C, with a single larger step of mass loss at 210 °C. The degradation profile differs significantly from the azide one, which features 3 steps starting at 108 °C, 223 °C, and 344 °C. These results correlate with the proposed click reaction of the azide from the polymer and the alkyne bond of the cobaltocenium compound.

### 3.4 Click reaction using ethynylferrocene 10

**Fig. 6:**
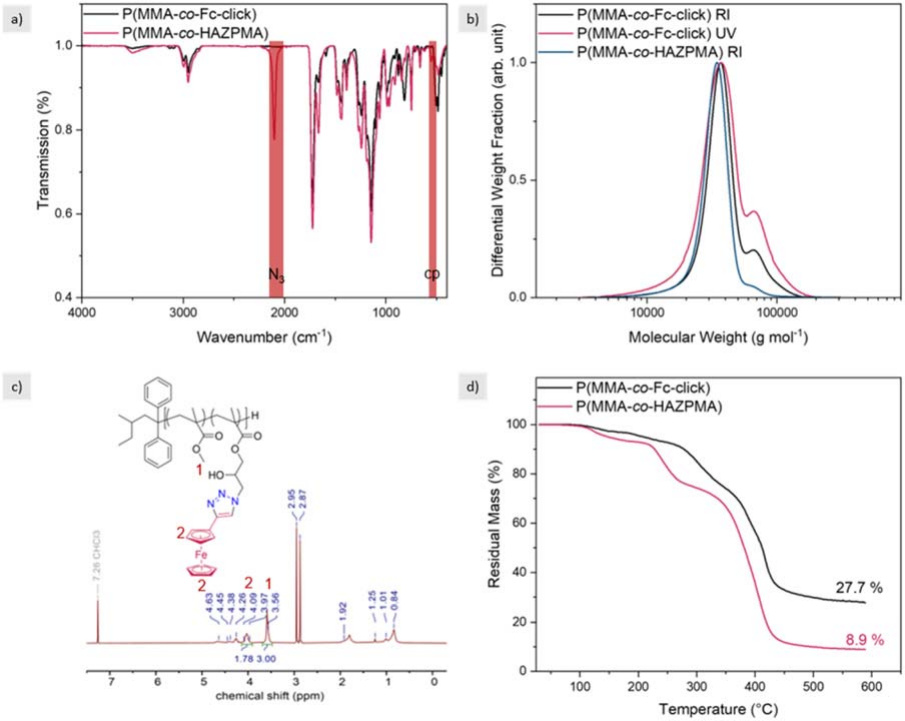
FTIR spectrum of the click reaction using ethynyl ferrocene (a), SEC data measured in DMF against PMMA standard (b), ^1^H-NMR spectrum measured in CDCl_3_ at 400 MHz (c) and TGA measurement under nitrogen atmosphere (d).

As previously shown, the azidated GlyMA polymer underwent a click reaction with the cobaltocenium compound; the same reaction was performed using ethynylferrocene 10 (Scheme 5). The reaction conditions were left unchanged, using a DMF/water (90/10, V/V) mixture as the solvent and 0.2 eq. CuBr_2_ as the copper source and 4 eq. sodium ascorbate as the reducing agent to generate the Cu(i) species. As observed previously, the azide valence vibration in the IR spectrum after the click reaction was no longer visible, indicating that the reaction had taken place. Fig. 6a shows these results with the vibration of the Cp ring of the ferrocene occurring at 557 cm^−1^. By the absence of the azide vibrations, complete conversion of the educt can be assumed. Based on the SEC traces measured in DMF (Fig. 6b), an increase in molecular weight is observed, as expected. The peak shifts to higher molecular weights, and the UV detector shows a strong signal that matches the RI detector, suggesting a number average molecular weight of 34.7 kDa with a dispersity of *Ð* = 1.20. In the SEC traces, a small shoulder at high molecular weights is observed, which explains the increase in dispersity. What exactly causes this shoulder is not known, as it is only observed in this reaction and not in the one with the cobaltocenium. It could be attributed to crosslinking reactions of unopened epoxide groups, but this is more likely at elevated temperatures rather than at room temperature. The protons of the aromatic system of the ferrocene can be observed in the ^1^H-NMR (Fig. 6c) at 3.97–4.09 ppm, similar to the NMR spectrum of the direct ring opening of the epoxide with 3-ferrocenylpropyl amine (Fig. 3c). The ceramic yield of the polymer also increased as expected. The TGA gives an increase of 18.8% compared to the azide-functionalized polymer. This resulted in a residual mass of 27.7 mass% at 600 °C in a nitrogen atmosphere, as concluded from Fig. 6d. The ceramic yield is 7.2 mass% higher than the functionalization of the direct opening with 3-ferrocenyl propylamine 8, which indicates a higher degree of functionalization.

### 3.5 Cyclic voltammetry

**Fig. 7:**
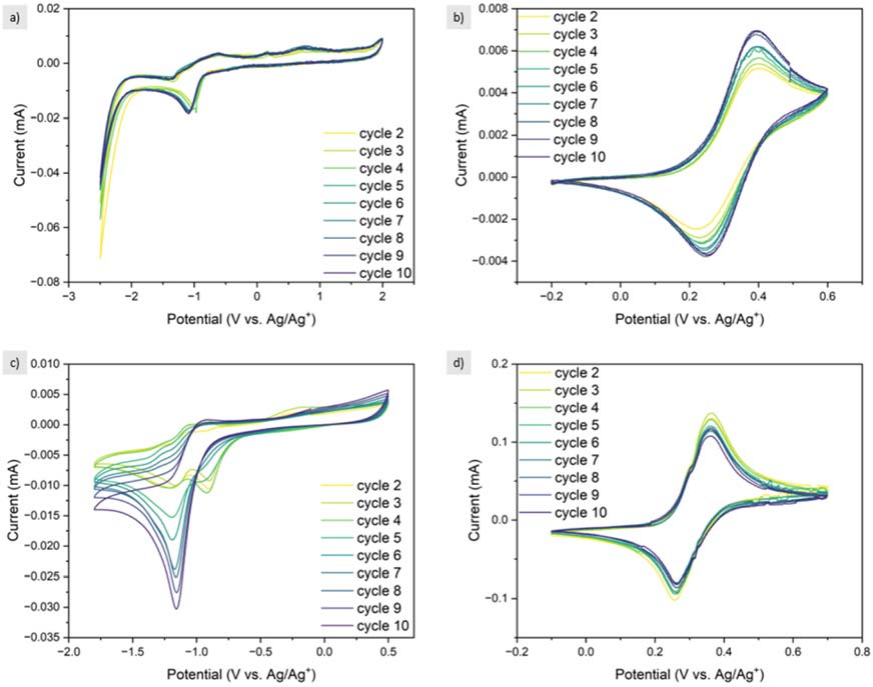
Cyclic voltammetry measurements of P(MMA-*co*-2-AAQ) 5 (a), P(MMA-*co*-FcAmine) 6 (b), P(MMA-*co*-Co-click) 3 (c) and P(MMA-*co*-Fc-click) 4 (d). All measurements were performed in acetonitrile with 0.1 M tetrabutylammonium hexafluorophosphate (TBA[PF_6_]) and a scanning rate of 50 mV s^−1^.

The previously discussed post-modifications of either the GlyMA or its azidated form, HAZPMA, with different metallocenes and 2-aminoanthraquinone all feature an electrochemically addressable group. Therefore, CV measurements were performed in order to address these functional groups. For the measurements, acetonitrile was chosen as the solvent because it has a wide potential window during analysis and does not undergo electrolysis. All samples were measured in a 0.1 M solution of tetrabutylammonium hexafluorophosphate with a scan rate of 50 mV s^−1^. For better comparison, cyclic voltammetry measurements of the unmodified polymers and the four redox-active small molecules were measured and are shown in Fig. S6. For the unmodified polymers, no redox activity can be observed, as expected (Fig. S6a and Fig. S6b). Starting with the 2-aminoanthraquinone-modified polymer 5, the CV (Fig. 7a) shows a reduction peak at −1.01 V *vs.* Ag/Ag^+^. This reduction is only partially reversible, as the corresponding oxidation peak is low-intensity at −0.53 V. This behaviour of 2-AAQ is also reported in the literature.^33,49^ For anthraquinones two separated one electron reduction processes would be expected with each having its corresponding re-oxidation process, which can be observed when the 2-AAQ is measured alone (Fig. S6c). The voltage of the oxidation peak in the polymer is shifted to higher voltages as compared to the single 2-AAQ, where it can be observed at −1.12 V *vs.* Ag/Ag^+^. The substitution with an amino group, which has a +M effect, increases the electron density in the ring system. As a consequence, this leads to a cathodic shift of the reduction potential compared to the unsubstituted anthraquinone.^49^ Especially for the 2-amino substituted anthraquinone, the second reduction feature is less reversible; side reactions like the oxidation reactions of the nitrogen can happen due to the increased electron density on the ring. The polymer containing the 3-ferrocenyl propylamine, which was used to open the epoxide ring, showed great reversible reduction, and oxidation was observed (Fig. 7b). The reduction peak is located at 0.25 V with the corresponding oxidation peak at 0.40 V against Ag/Ag^+^. The single 3-ferrocenyl propylamine 8 shows the reduction at −0.03 V and the oxidation at 0.13 V against Ag/Ag^+^, indicating a shift of the reduction and oxidation of the ferrocene unit to higher voltages when bound to the polymer (Fig. S6e). Comparing the voltages of the reduction and oxidation of the ferrocene with the voltages of the sample (Fig. 7d) originating from the azide-modified polymer *via* the click reaction, which also contains ferrocene as the switchable unit, the reduction is observed at 0.26 V with the oxidation taking place at 0.36 V against Ag/Ag^+^. The reduction potential is shifted by a small amount of 0.1 V, whereas the oxidation of the ferrocene is shifted by 0.4 V to a lower potential. This could be due to the different chemical environment of the ferrocene, as in the ferrocene unit from the click reaction, the triazole moiety is in direct proximity to the switchable group. When compared to the single ethynylferrocene 10, which is shown in Fig. S6f, the reduction peak can be observed at 0.17 V with the corresponding oxidation taking place at 0.37 V. When bound to the polymer *via* the triazole system, the oxidation voltage is shifted by 0.01 V to higher voltages and the reduction voltage by 0.09 V to lower voltages. Both samples containing the ferrocene show reversible switchability over multiple cycles. Leading to a stimuli-responsive metallopolymer. Due to solubility issues, the samples from the click reactions were not measured in solution. The samples were mixed with multi-walled carbon nanotubes at a 2 : 1 weight ratio and suspended in chloroform. This mixture was then used to drop-coat a glassy carbon electrode as the working electrode. Looking at the cobaltocene-containing polymer after the click reaction (Fig. 7c), cyclic voltammetry shows two reduction peaks in the first cycles at −0.92 V and −1.16 V. With increasing number of cycles, the peak at −0.92 V disappears, and the reduction signal at −1.16 V becomes more intense until after cycle 6 the peak at −0.92 V is not observable anymore. Comparing this to an earlier work of our group, this disappearing signal can also be seen with a small intensity.^48^ An oxidation peak at −0.92 V is visible in the latest cycles. In the first few cycles, small oxidation peaks at −1.25 V and −1.05 V can be observed, which disappear over time.^48^ When compared to the ethynylcobaltocenium hexafluorophosphate 9 itself, the reduction peak, which is visible at −1.16 V after 10 cycles, is observed with a small shift to −1.12 V for the pure ethynylcobaltocenium hexafluorophosphate 9 (Fig. S6e). The oxidation peak for the pure compound is located at −1.03 V, which could correspond to the oxidation peak at −1.06 V for the functionalized polymer. The observation that two reduction peaks are visible in the beginning and, with increasing number of cycles, one peak decreases, whereas the other one increases could be due to two electrochemically distinguishable cobaltocene environments. The gradual decrease of one peak and the increase of the other one with increasing number of cycles suggests a change in relative population of the two environments. This may arise from electrochemically induced structural reorganization of the polymer coating on the electrode or changes in ion transport within the coating. Similar observations of cycling-induced changes in the morphology of poly(vinylferrocene) films have been reported earlier in the literature.^50^

## 4 Conclusions

In this work, we have demonstrated the successful anionic polymerization of copolymers containing glycidyl methacrylate (GlyMA). Owing to the high reactivity of the epoxide functionality, carefully optimized polymerization conditions were required to suppress undesired ring-opening reactions and crosslinking. The resulting polymers exhibited narrow molar mass distributions, low dispersities, and well-defined architectures, highlighting the control of anionic polymerization to provide access to functional polymer materials.

The incorporation of GlyMA offers a versatile platform for post-polymerization modification. Direct ring-opening reactions of epoxide groups with electrochemically active amines, including 3-ferrocenylpropyl amine and 2-aminoanthraquinone, were achieved to moderate degrees of functionalization and characterized by NMR, SEC, FTIR, DSC, and TGA, thereby demonstrating the feasibility of the post-modification strategy. Furthermore, conversion of the epoxide groups into azides paved the way for subsequent click-functionalization strategies. Click reactions with metallocene derivatives such as ethynylferrocene and especially ethynylcobaltocenium hexafluorophosphate enabled the introduction of additional redox-active functionalities, generating multiple redox-switchable platforms from one base polymer. The electrochemical switchability of the resulting polymers was confirmed by cyclic voltammetry, demonstrating the stimuli-responsive nature of the incorporated functional groups. Overall, the synthetic strategy developed in this work provides a robust and flexible route to precision-functionalized polymer materials. The ability to combine controlled anionic polymerization with versatile post-functionalization significantly expands the design space for advanced polymer architectures and establishes a promising foundation for future responsive materials. In particular, the electroactive polymers developed here offer considerable potential for application in electrochemically mediated separations^51,52^ and emerging carbon capture strategies,^33,53^ as previously shown with the cobaltocenium hexafluorophosphate.^47^

## Author contributions

The manuscript was written through contributions of all authors. Experimental design: S.H., V.G., M.G.; monomer and polymer synthesis: S. H., V. G.; performance of experiments: S. H., for homopolymer V.G.; data analysis: S. H.; writing of the first manuscript draft: S. H.; writing of the manuscript: S.H., M.G.; all authors have given approval to the final version of the manuscript.

## Conflicts of interest

There are no conflicts to declare.

## Supplementary Material

RA-OLF-D6RA06652F-s001

## Data Availability

The data that support the findings of this study are available from the corresponding author upon reasonable request. Supplementary information (SI): Fig. S1: photographs of the positive Preussmann reaction showing the intact epoxide in the polymer, with the corresponding scheme. Fig. S2: SEC data of PGlyMA^16^ measured in DMF against PMMA standard. Fig. S3: ^1^H-NMR spectrum of PGlyMA^16^ measured at 400 MHz in CDCl_3_. Fig. S4: SEC measurement of the AAQ functionalized polymer measured in DMF against PMMA standard (a), TGA measurement in nitrogen atmosphere to 600 °C (b), DSC measurement (c) and ^1^H-NMR spectrum of the 2-AAQ functionalized polymer measured at 400 MHz in CDCl_3_ (d). Fig. S5: SEC measurement of P(MMA_70_-*co*-GlyMA_30_)^25^ and P(MMA-*co*-HAZPMA) in TFE against PMMA standard. Fig. S6: cyclic voltammetry measurements of P(MMA-*co*-GlyMA) 1 (a), P(MMA-*co*-HAZPMA) 2 (b), 2-AAQ 7 (c), 3-ferrocenyl propylamine 8 (d), ethynylcobaltocenium hexafluorophosphate 9 (e) and ethynylferrocene 10 (f). All measurements were performed in acetonitrile with 0.1 M tetrabutylammonium hexafluorophosphate (TBA[PF6]) and a scanning rate of 50 mV s^−1^. See DOI: https://doi.org/10.1039/d6ra06652f.
