# Supplementary material for: Tailoring stimuli-responsive copolymers via a universal poly(glycidyl methacrylate) modification strategy
Source: RSC Adv. 2026 Aug 14;16(41):48183–93. doi: 10.1039/d6ra06652f (PMC13474308; doi:10.1039/d6ra06652f)
Supplement: Supplementary file 1 [file RA-016-D6RA06652F-s001.pdf]

Supplementary Information (SI) for RSC Advances.
This journal is © The Royal Society of Chemistry 2026

Supporting Information

Tailoring Stimuli-Responsive Copolymers via a Universal Poly(glycidylmethacrylate) Modification Strategy

Sebastian Heinz[1], Victoria Glößner[1], Markus Gallei [1,2,]*

[1]Saarland University, Polymer Chemistry, Campus C4 2, 66123 Saarbrücken, Germany.

[2]Saarene, Saarland Center for Energy Materials and Sustainability, Campus C4 2, 66123 Saarbrücken, Germany.

[*]Corresponding author

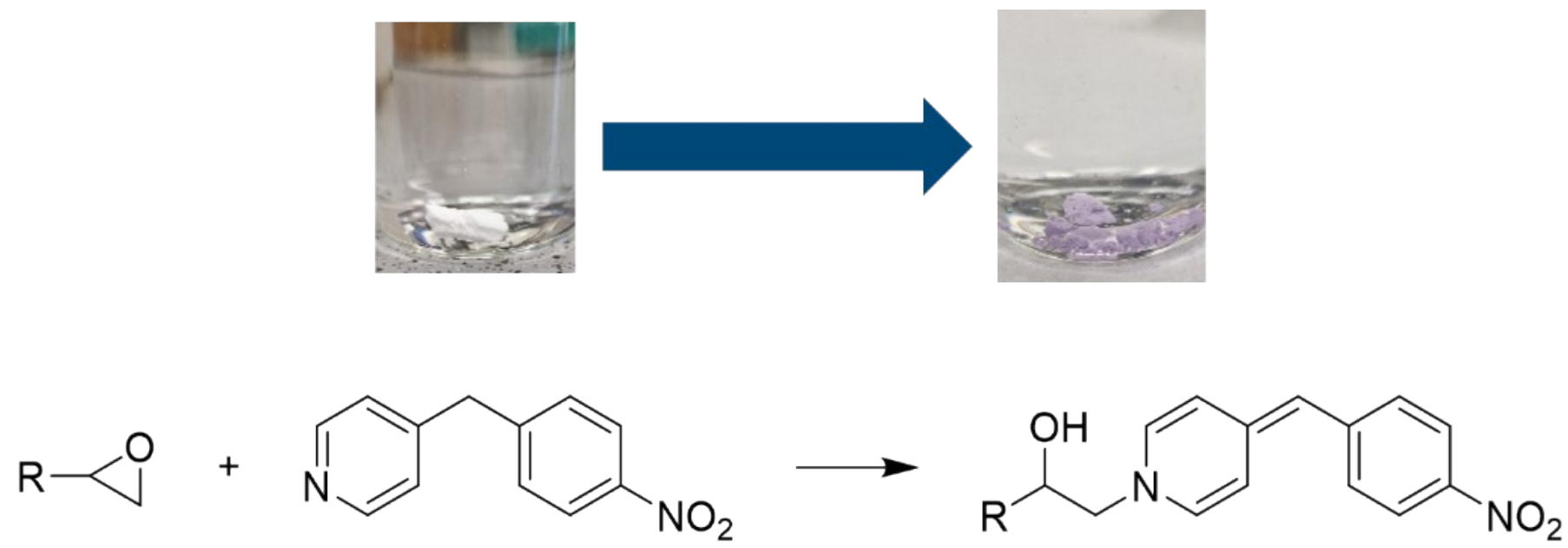

Figure S1 Photographs of the positive Preußmann reaction showing the intact epoxide in the polymer, with the corresponding scheme.

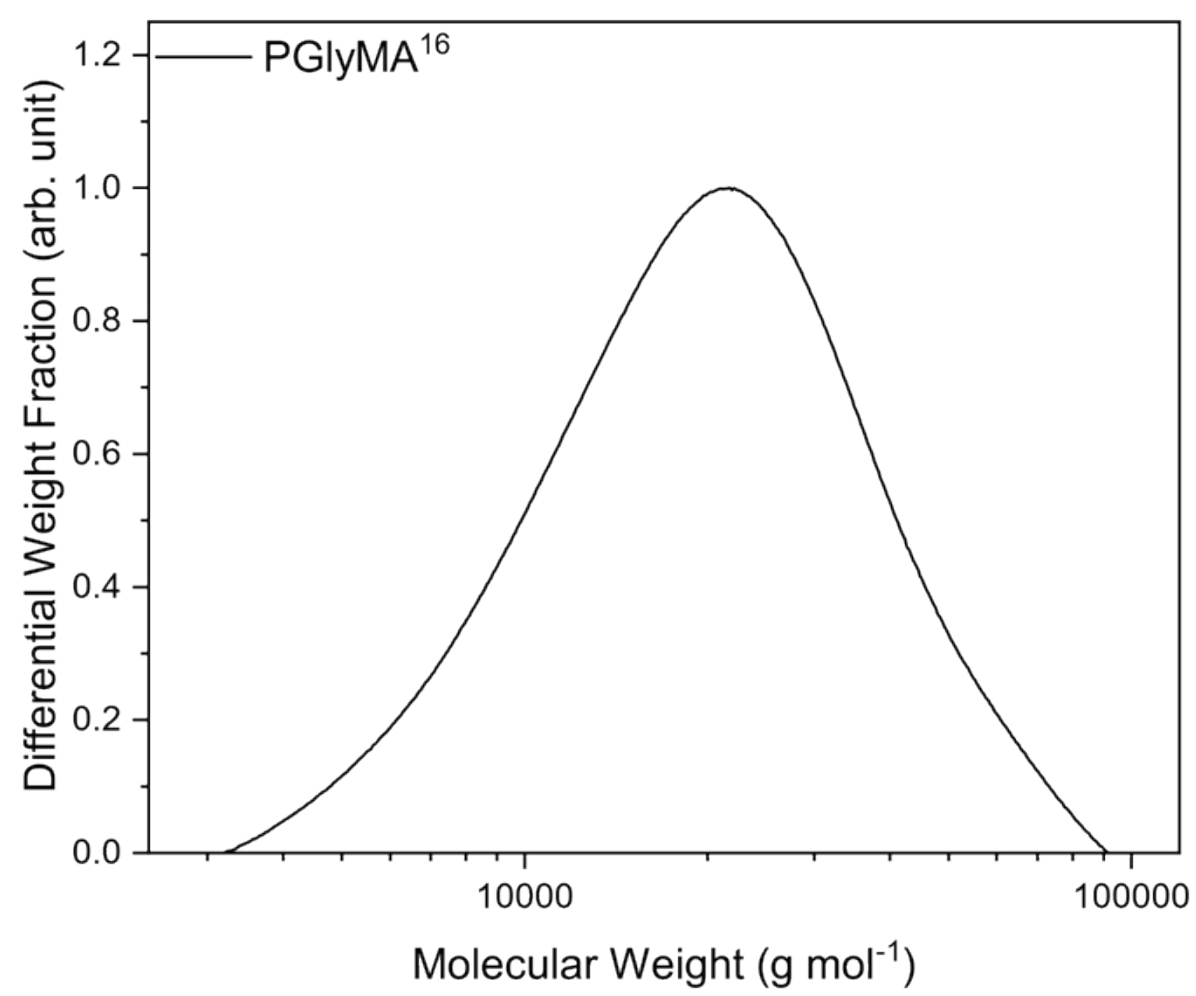

Figure S2 SEC data of PGlyMA[16] measured in DMF against PMMA standard.

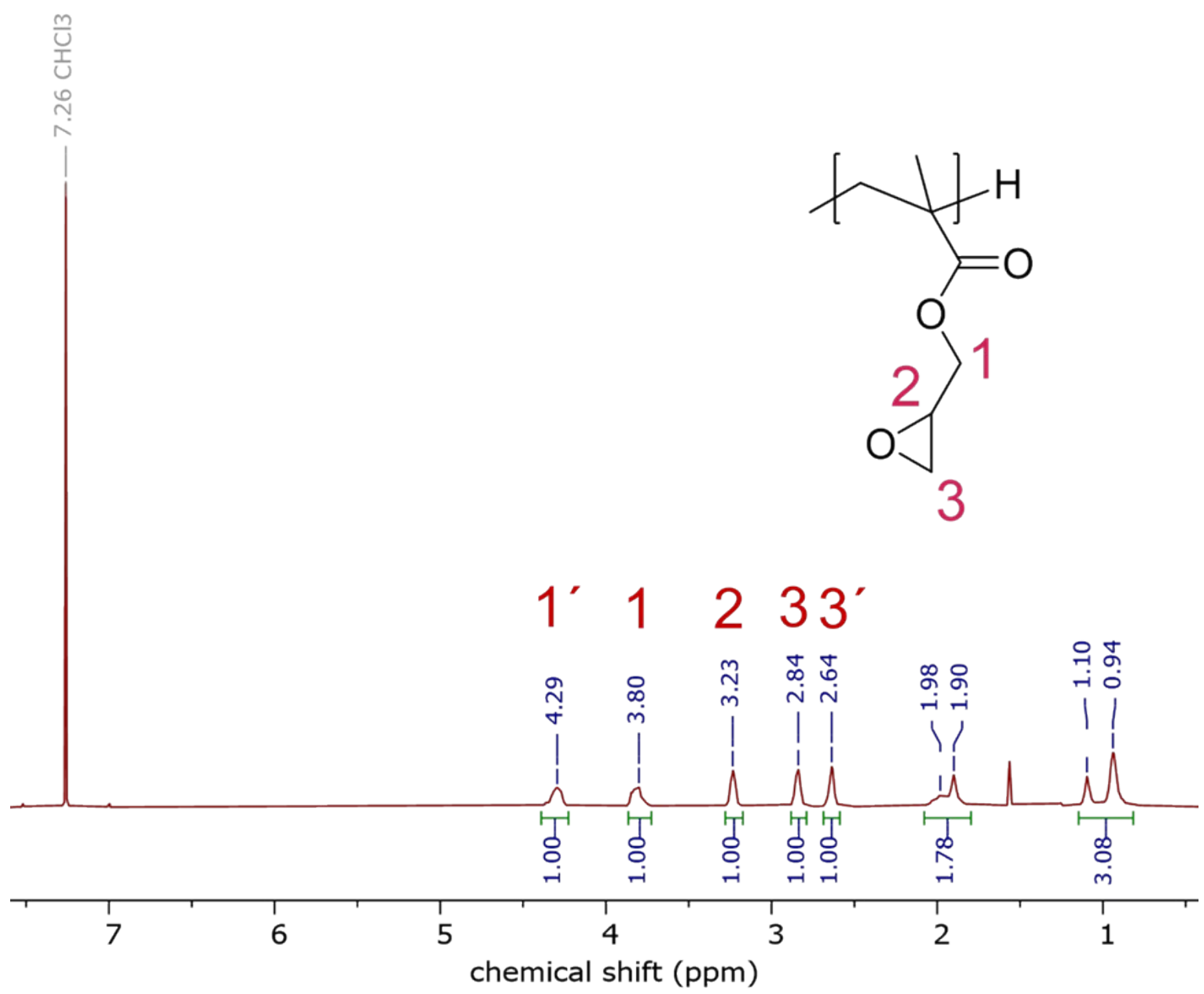

Figure S3 ^{1}H-NMR spectrum of PGlyMA measured at 400 MHz in $CDCl_3$.

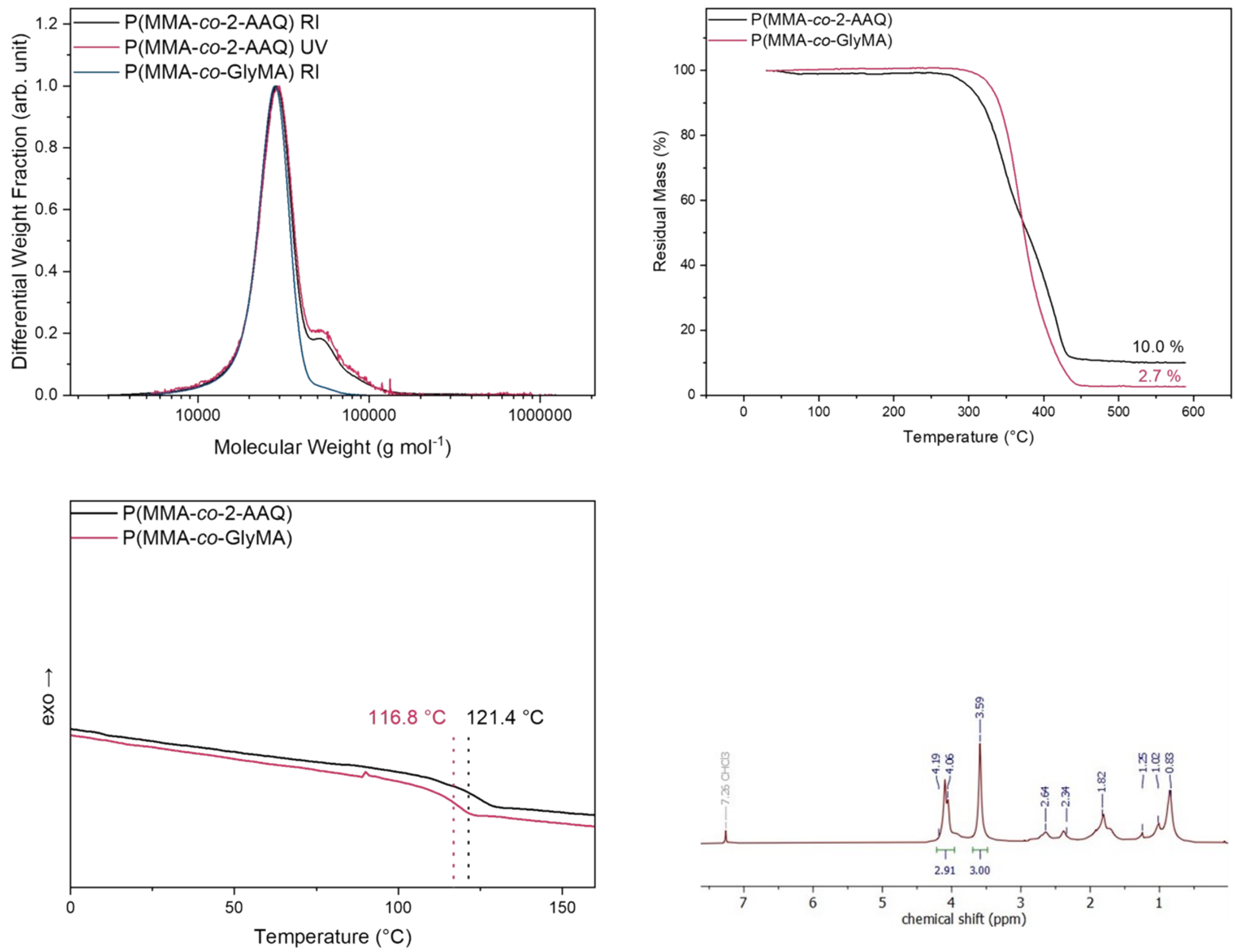

Figure S4 SEC measurement of the AAQ functionalized polymer measured in DMF against PMMA standard (a), TGA measurement in nitrogen atmosphere to 600 °C (b), DSC measurement (c) and ^{1}H-NMR spectrum of the 2-AAQ functionalized polymer measured at 400 MHz in $CDCl_3$ (d).

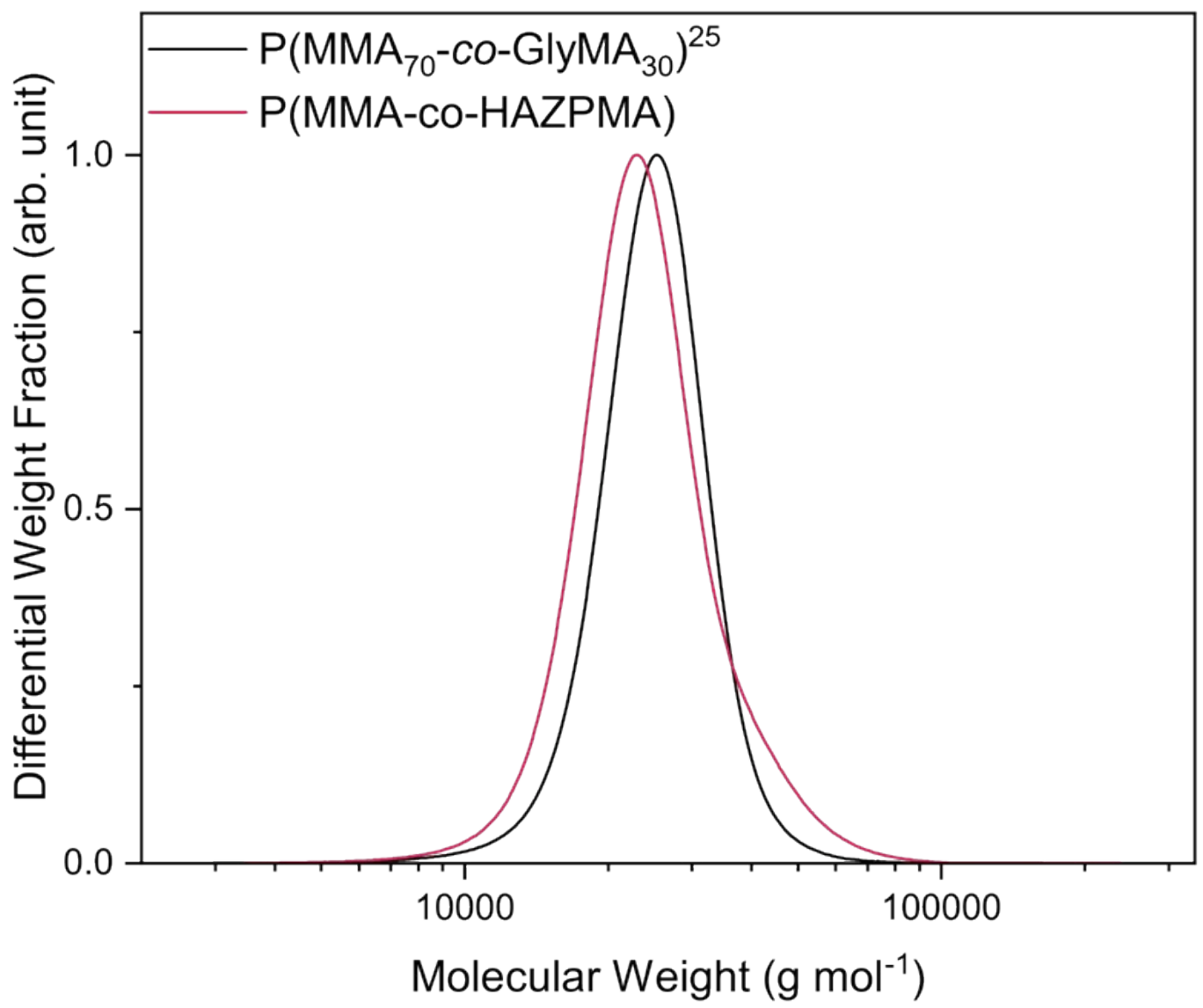

Figure S5 SEC measurement of P(MMA$_{70}$-*co*-GlyMA$_{30}$)[25] and P(MMA-*co*-HAZPMA) in TFE against PMMA standards.

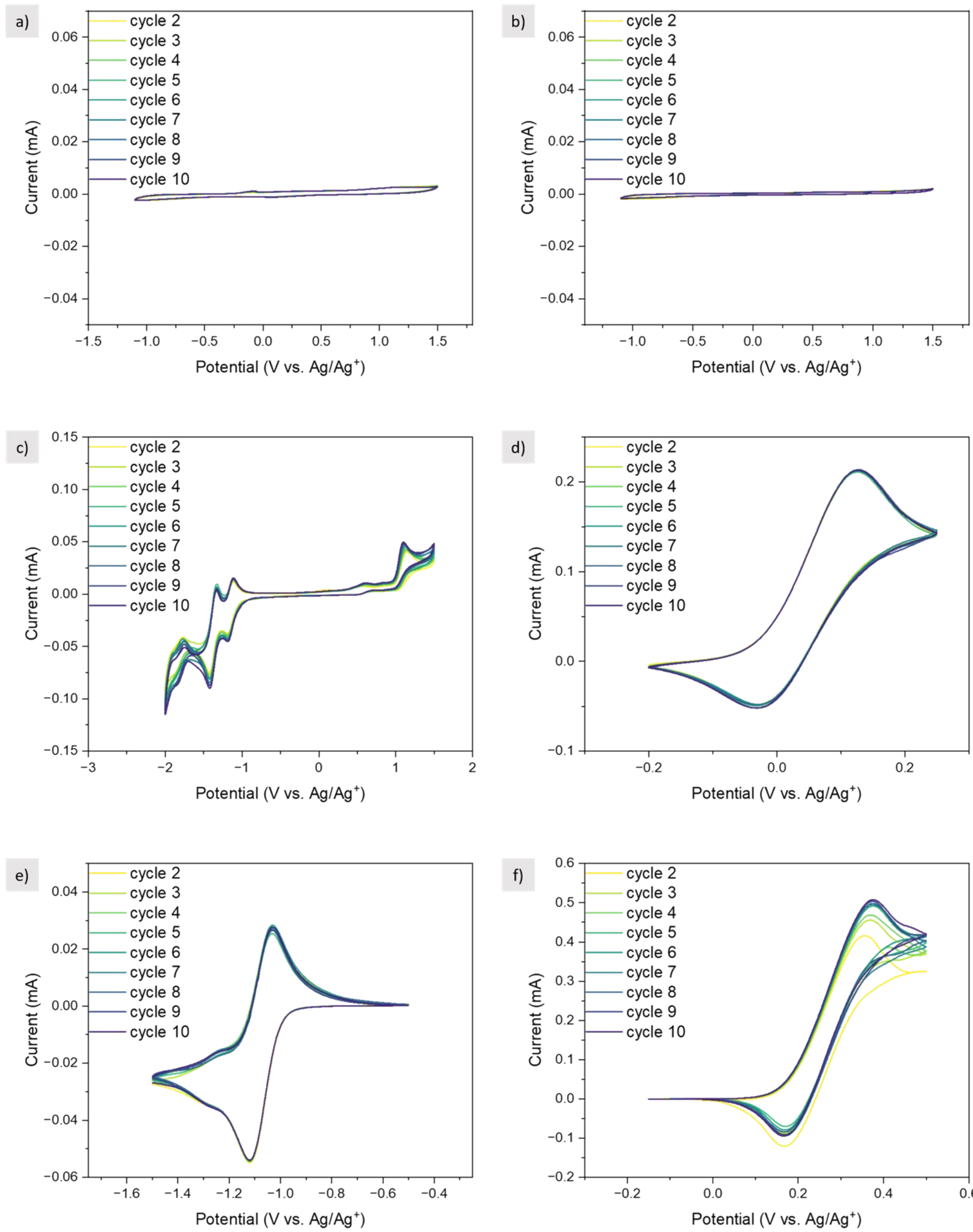

Figure S6 Cyclic voltammetry measurements of P(MMA-*co*-GlyMA) **1** (a), P(MMA-*co*-HAZPMA) **2** (b), 2-AAQ **7** (c), 3-ferrocenyl propylamine **8** (d), ethynylcobaltocenium hexafluorophosphate **9**

(e) and ethynylferrocene **10** (f). All measurements were performed in acetonitrile with 0.1 M tetrabutylammonium hexafluorophosphate (TBA[PF_6]) and a scanning rate of 50 mVs^{-1}.
